# COVID-19 vaccines breakthrough infection and adverse reactions in medical students: a nationwide survey in Iran

**DOI:** 10.3389/fpubh.2024.1348015

**Published:** 2024-03-13

**Authors:** Amirreza Allahgholipour, Seyed Amir Ahmad Safavi-Naini, Zahra Shekarriz Foomany, Abdolvahab Eskandari, Hossein Nazari Rostami, Mohamad Javad Honarvar, Mohammad Mohammadi, Parnian Khalili, Mehran Ilaghi, Hossein Afshar, Ali Amini Baghbadorani, Hamid Reza Moghimi, Alireza Chamani Goorabi, Amirreza Mehrparvar, Mehdi Safari, Ashraf Sadat Nakhli, Mohammad Mahmoudabadi, Adib Seifadini, Sobhan Sheikhansari, Sadaf Khojastehfar, Parisa Mahdavi, Maede Mohammadi, Siyamak Ashrafi Barzideh, Nadia Akbarzadeh, Seyed Hosein Delavarpour Moghadam, Ali Tavakoli Pirzaman, Mohammad Barary, MohammadAli Emamhadi

**Affiliations:** ^1^Student Research Committee, School of Medical Education and Learning Technologies, Shahid Beheshti University of Medical Sciences, Tehran, Iran; ^2^Gastroenterology and Liver Diseases Research Center, Research Institute for Gastroenterology and Liver Diseases, Shahid Beheshti University of Medical Sciences, Tehran, Iran; ^3^Student Research Committee, School of Medicine, Shahid Beheshti University of Medical Sciences, Tehran, Iran; ^4^Endocrine Research Center, Research Institute for Endocrine Sciences, Shahid Beheshti University of Medical Sciences, Tehran, Iran; ^5^Student Research Committee, Shahid Sadoughi University of Medical Sciences, Yazd, Iran; ^6^Student Research Committee, Shiraz University of Medical Sciences, Shiraz, Iran; ^7^Institute of Neuropharmacology, Kerman Neuroscience Research Center, Kerman University of Medical Sciences, Kerman, Iran; ^8^Student Research Committee, School of Rehabilitation, Shahid Beheshti University of Medical Sciences, Tehran, Iran; ^9^Student Research Committee, School of Public Health and Safety, Shahid Beheshti University of Medical Sciences, Tehran, Iran; ^10^Student Research Committee, Faculty of Nutrition and Food Technology, Shahid Beheshti University of Medical Sciences, Tehran, Iran; ^11^Student Research Committee, Babol University of Medical Sciences, Babol, Iran; ^12^Department of Forensic Medicine, School of Medicine, Shahid Beheshti University of Medical Sciences, Tehran, Iran

**Keywords:** COVID-19, COVID-19 vaccines, COVID-19 breakthrough infections, drug-related side effects and adverse reactions, vaccination

## Abstract

**Introduction:**

There are different types of COVID-19 vaccines approved worldwide. Since no national studies focus on vaccine-related adverse reactions and breakthrough cases, this study aimed to investigate the rate of adverse events and COVID-19 infection in medical students in Iran.

**Methods:**

This retrospective cohort study included Iranian medical students who received two doses of COVID-19 vaccines. The medical team gathered the demographic characteristics, comorbidities, type of vaccine, adverse events following vaccination, and history of COVID-19 infection data through a phone interview. The frequency of adverse events and breakthrough infection was stratified by vaccine type (ChAdOx1-S, Gam-COVID-Vac, and BIBP-CorV).

**Results:**

A total of 3,591 medical students enrolled in this study, of which 57.02% were females, with a mean age of 23.31 + 4.87. A PCR-confirmed and suspicious-for-COVID-19 breakthrough infection rate of 4.51 and 7.02% was detected, respectively. There was no significant relation between breakthrough infection and gender, BMI, blood groups, and comorbidities. However, there was a significant difference in breakthrough infection rate among different types of vaccines (*p* = 0.001) and history of COVID-19 infection (*p* = 0.001). A total of 16 participants were hospitalized due to COVID-19 infection after vaccination for reasons such as dyspnea, abnormal imaging, or decreased oxygen saturation. No severe infection or death was observed in the studied population.

**Conclusion:**

Vaccination prevented severe COVID-19 infection, although a high breakthrough infection rate was evident among Iranian medical students during the Delta variant’s peak. Vaccine effectiveness may be fragile during emerging new variants and in high-exposure settings. Moreover, adverse events are rare, and the benefits of vaccination outweigh the side effects. However, many limitations challenged this study, and the results should be cautious.

## Introduction

The rapid spread of the coronavirus disease 2019 (COVID-19) caused 235 million infected cases and 4.8 million lives claimed globally (1). Many countries decided to use COVID-19 vaccines under Emergency Use Authorization (EUA) to prevent further fatality and disruption caused by the pandemic (2, 3). COVID-19 vaccines dramatically reduced the disease’s mortality; some concerns were raised regarding their safety and effectiveness (4). The studies conducted in Iran on the efficacy and side effects of vaccines have mostly been on BIBP-CorV (Sinopharm), ChAdOx1-S (Oxford–AstraZeneca), Gam-COVID-Vac (Sputnik V), and BBV152 (Covaxin) vaccines, which is due to the availability of these vaccines in our country (5–8).

According to the World Health Organization (WHO), BIBP-CorV is a β-propiolactone-inactivated vaccine, which can be stored at 2–8°C and administered on a 0/21–28-day schedule, with 79.34% effectiveness. To increase immunogenicity, the vaccine contains aluminum hydroxide as an adjuvant (9, 10). As of December 31, 2020, it is approved by the National Medical Products Administration of China and about 45 countries/jurisdictions for adults 18 years and older (11). It was proven to be a safe vaccine and can be administered in special populations, like patients with multiple sclerosis (MS), based on prior inactivated vaccine safety in MS patients and Sinopharm’s lack of severe adverse effects in the primary trial phase (7). However, several adverse effects have been reported, including pain around the injection site, fatigue, fever, headaches, and lethargy (10).

On August 11, 2020, the Russian Ministry of Health released Gam-Covid-Vak, developed by the Gamalia Institute of Epidemiology and Microbiology. According to the preliminary analysis of the human phase 3 trials published on February 2, 2021, the vaccine worked with 91.6% effectiveness. It is an adenovirus carrier vaccine. Most adenovirus vaccines have demonstrated a positive immune response (cellular and humoral immunity) after the first shot. As a result of the second dose of vaccine injection, stronger immunity, and a longer duration have been observed (10, 12, 13). For the first and second vaccinations of the vaccine, Russian scientists devised a unique idea to increase its effectiveness by using two different types of adenovirus vectors (rAd26 and rAd5), which were successful in both phases of clinical trials. Despite the vaccine being stored and distributed at −18°C, an optimal temperature profile for global distribution is achieved between 2 and 8°C (10). In Iran, the first phase of the COVID-19 vaccination program for healthcare workers was launched on February 9, 2021, with the Sputnik V COVID-19 vaccine (6).

The ChAdOx1-S vaccine, a virus-vector vaccine, can demonstrate an efficacy of about 66.7% over the 14-day course after the second injection (14). There are a few side effects after the injection, including nausea, malaise, headache, and injection-site reaction and pain. Nonetheless, all these everyday adverse events usually resolve after a few days. Few studies have reported severe vaccine-related adverse events like capillary leak syndrome, cutaneous vasculitis, systemic sclerosis, and Vaccine-Induced Immune Thrombotic Thrombocytopenia (VITT) following injection (15–17).

Covaxin, manufactured by Bharat Biotech (India), is a Vero cell-based whole-virion inactivated SARS-CoV-2. The vaccine has been formulated with imidazoquinoline gallamide (IMDG), an agonist for toll-like receptor 7/8 (18). It showed 77.8% effectiveness in symptomatic cases, resulting in 65.2% protection against the Delta variant (19). Nausea/vomiting, fever, fatigue, headache, and injection-site pain were the most frequent adverse events (18).

Studies have shown various local or systemic side effects induced by COVID-19 vaccines (20), for instance, induration, tenderness, erythema, and, infrequently, near-injection-site abscess. On the other hand, cough, headache, fever, and chills constitute systemic reactions. Moreover, anaphylaxis has been reported as an uncommon reaction (21). Some side effects have never been registered with previous vaccines, like VITT, a hyper-thrombotic condition (22). Further, the increasing number of reports worldwide about acute myocarditis induced by the mRNA COVID-19 vaccine raised significant concerns (23).

It has to be stated that the adverse events are mild and self-limiting in most cases (24). However, COVID-19 vaccines are under emergency use approval, and some adverse reactions may remain obscure. Anaphylaxis can occur after vaccination and has a reporting rate of about 1 case in 1 million, but it gets much attention due to its severity and importance (24). Also, vaccines passed different regulations for approval, and types administrated in developed countries are more closely investigated in the literature than in developing countries. Therefore, surveillance programs are needed better to evaluate the adverse events of the COVID-19 vaccines, especially in developing countries.

In addition to the fact that COVID-19 vaccines do not provide complete protection, individuals are also at risk of post-vaccination breakthrough infection due to the development of mutations and different variants of SARS-CoV-2.

Previous studies reached precious findings about COVID-19 prevalence and COVID-19 vaccines in diverse populations. A systematic review by Zheng et al. demonstrated that, compared to healthcare workers (HCWs) and the general population, the older adults have the lowest rate of vaccination effectiveness, 83.8%. Meanwhile, the general population and HCWs showed 86.1 and 95.3% effectiveness, respectively (25). Moreover, another study revealed that HCWs suffer a higher prevalence of SARS-CoV-2 infection compared to the general population (26, 27). Medical and paramedical students seem to be missing from this puzzle. Especially when there is a significant vaccine hesitancy among this group (approximately 25%) (28), and they are at the age that social media significantly influences them (29).

Still, there is a paucity of evidence regarding the safety and efficacy of vaccines in large populations. Since no national studies focus on vaccine-related adverse reactions and breakthrough cases, this study aimed to investigate the rate of adverse events and COVID-19 infection in medical students in Iran.

## Materials and methods

### Ethics statement

The Institutional Review Board of Shahid Beheshti University of Medical Sciences approved this study protocol (reference code: IR.SBMU.RETECH.REC.1400.947). Informed consent was obtained through phone calls, and confidentiality of data was a concern during the study. All interventions were performed following the Declaration of Helsinki.

### Study design

In this retrospective cohort study conducted from August 2021 to February 2022, medical students who received two doses of COVID-19 vaccines were enrolled from 5 provinces (Tehran: 2899, Shiraz: 333, Kerman: 121, Yazd: 74, Hamedan: 31, Other: 133). Subjects’ information, including their demographic data, comorbidities, history of COVID-19 infection (before, after, and during (the first to 14 days after receiving the second dose) immunization period), blood group, body mass index (BMI), history of influenza vaccination, and adverse events following immunization was gathered by a medical team via a follow-up call from August 28 to September 22, 2021 (the delta variant was the dominant strain of the virus at that time). Participants with two unanswered phone calls with a minimum of 24-h intervals between calls and subjects receiving two vaccine types were excluded. The study’s outcome was a breakthrough infection rate among medical students after full immunization and adverse reactions to COVID-19 vaccines.

### Definitions and grades

The method of COVID-19 diagnosis was based on self-reporting during the call and then verifying the provided information with the database provided by the health authorities. The study recorded and analyzed the diagnostic method of patients whose information was confirmed using the database provided by the health authorities. Moreover, subjects with COVID-19 symptoms but who did not undergo PCR tests (COVID-19 suspected patients) were also included. Iran’s Ministry of Health defines Suspicious-for-COVID-19 as a patient with respiratory symptoms, exposure to COVID-19, and no other robust explanation for the symptoms. In the case of two negative PCR tests, the COVID-19 infection was ruled out. Categories of severity for COVID-19 infection were as follows: Mild: any sign of COVID-19 infection without any dyspnea or abnormal imaging; moderate: patient experiencing dyspnea or hospitalization or abnormal imaging; severe: decrease in oxygen saturation (<94%), or intensive care unit admission (30). A severe adverse event after vaccination is a side effect that needs hospitalization for further evaluation or treatment.

### Statistical analysis

R software version 3.6.3 was used for the statistical analysis. Descriptive statistics were provided as mean ± standard deviation or number and percentage. Mann–Whitney U test was used to evaluate the difference between the means of the two groups. Categorical variables were compared using the chi-square test. A value of *p* <0.05 was considered significant.

## Results

A total of 3,591 medical students with a mean age of 23.31 ± 4.87 years, 57.02% of whom were female, were investigated. Mean BMI was 22.9 + 3.71 kg/m^2^, and O, A, B, and AB blood groups were evident in 35.85, 31.7, 22.48, and 10% of students, respectively. Of 180 reported comorbidities, asthma (31) and thyroid gland disorders (26) were among the most frequent underlying diseases. All the patient’s demographic information and their history of COVID-19 infection are given in Table 1.

**Table 1:** Baseline characteristics of patients among different groups of vaccines.

Variables	COVID-19 vaccine, *n* (%)			
	ChAdOx1-S	Gam-COVID-Vac	BIBP-CorV	All vaccines
Age^1^, years	23.35 ± 4.48	23.68 ± 4.83	22.89 ± 5.04	23.31 ± 4.87
Gender				
Male	420 (45.9)	525 (44.5)	563 (39.7)	1,543 (43.0)
Female	496 (54.1)	656 (55.5)	855 (60.3)	2048 (57.0)
BMI^1^, kg/m^2^	23.13 ± 3.74	22.97 ± 3.70	22.71 ± 3.65	22.93 ± 3.71
Dose of injected vaccine				
1	69 (7.5)	29 (2.5)	131 (9.2)	246 (6.9)
2	847 (92.5)	1,152 (97.5)	1,287 (90.8)	3,345 (93.1)
Covid-19 infection before vaccination	274 (29.9)	340 (28.8)	404 (28.5)	1,042 (29.0)
Covid-19 infection after vaccination	67 (7.3)	122 (10.3)	140 (9.9)	334 (9.3)
Covid-19 infection frequency before vaccination				
Not Infected	619 (67.6)	824 (69.8)	968 (68.3)	2,458 (68.4)
1	259 (28.3)	306 (25.9)	401 (28.3)	990 (27.6)
> 1	38 (4.1)	51 (4.3)	49 (3.5)	143 (4.0)
Influenza vaccination in the past year	122 (13.3)	191 (16.2)	61 (4.3)	374 (10.4)
Blood group				
A	299 (32.6)	387 (32.8)	426 (30.0)	1,137 (31.7)
B	207 (22.6)	283 (24.0)	303 (21.4)	808 (22.5)
AB	80 (8.7)	108 (9.1)	163 (11.5)	359 (10.0)
O	330 (36.0)	403 (34.1)	526 (37.1)	1,287 (35.8)
Rh positive	793 (86.6)	1,031 (87.3)	1,234 (87.0)	3,119 (86.9)
Underlying disease history	44 (4.7)	69 (5.8)	67 (4.5)	180 (5.0)
Asthma	6 (0.7)	10 (0.8)	14 (1.0)	31 (0.9)
Thyroid disorder	8 (0.9)	11 (0.9)	6 (0.4)	26 (0.7)

^1^Age and BMI values are shown as mean ± SD.

**Figure 1:**
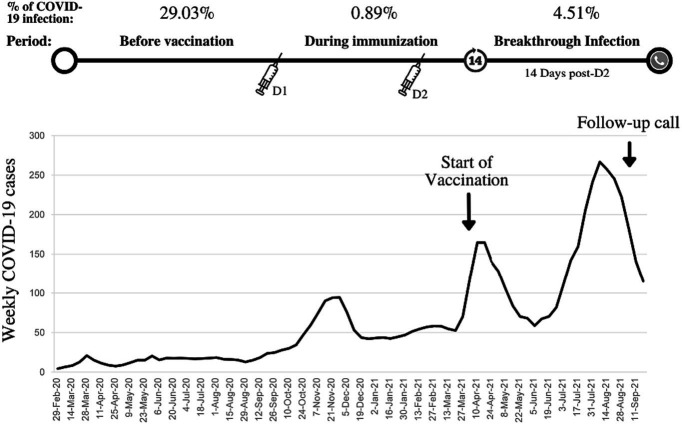
Weekly COVID-19 cases in Iran, the start of COVID-19 vaccination program in medical students, history of COVID-19 infection, and breakthrough infection rate (confirmed by polymerase chain reaction).

As depicted in Figure 1, a PCR-confirmed breakthrough infection rate of 4.51% was detected. In addition, the suspicious-for-COVID-19 breakthrough infection rate was 7.02%. In students injected with more than one dose of the COVID-19 vaccine, the breakthrough infection rate was lower than those infected with COVID-19 once before (1.55%, 2/129 vs. 2.32%, 23/990).

Table 2 describes the relationship between breakthrough infection and underlying factors and different types of COVID-19 vaccines. There was no significant relation between breakthrough infection and gender, BMI, blood groups, and comorbidities. However, there was a significant difference in breakthrough infection rate among different types of vaccines (*p* = 0.001), history of COVID-19 infection (*p* = 0.001), and history of influenza vaccination (*p* = 0.001). The severity of COVID-19 after vaccination is presented in Table 3, stratified by type of vaccine. The breakthrough rate was higher for ChAdOx1-S (6.22%) than for Gam-COVID-Vac (3.13%) and BIBP-CorV (4.23%) vaccines. A total of 16 participants were hospitalized for post-vaccination COVID-19 infection for reasons such as shortness of breath, abnormal imaging, or decreased oxygen saturation (up to 94%), as well as severe complications. All of them were PCR-positive.

**Table 2:** COVID-19 vaccine breakthrough in medical students.

Variables	COVID-19 infected^1^, *n* (%)	Not infected, *n* (%)	Value of *p*
Total	252 (7.0)	3,339 (93.0)	–
Gender			0.235
Female	145 (7.1)	1902 (92.9)	
Male	107 (6.9)	1,436 (93.1)	
BMI, kg/m^2^			0.717
<18.5	18 (5.2)	326 (94.8)	
18.5–24.9	170 (7.3)	2,155 (92.7)	
25–29.9	54 (7.0)	716 (93.0)	
>30	10 (6.6)	141 (93.4)	
Blood group			0.935
A	74 (6.5)	1,063 (93.5)	
AB	25 (7.0)	334 (93.0)	
B	58 (7.2)	749 (92.8)	
O	95 (7.4)	1,192 (92.6)	
Rh			0.285
Positive	227 (7.3)	2,892 (92.7)	
Negative	25 (5.3)	446 (94.7)	
History of COVID-19 infection			< 0.0001
Not Infected	218 (8.9)	2,240 (91.1)	
One time	31 (3.1)	960 (96.9)	
Two times	3 (2.6)	112 (97.4)	
Vaccine name			< 0.0001
Gam-COVID-Vac	56 (4.7)	1,125 (95.3)	
ChAdOx1-S	91 (10.0)	825 (90.0)	
BIBP-CorV	93 (6.6)	1,325 (93.4)	
COVIran Barekat	6 (12.5)	42 (87.5)	
BBV152	6 (22.2)	21 (77.8)	
Comorbidities			0.705
Yes	10 (5.6)	170 (94.4)	
No	242 (7.1)	3,168 (92.9)	
Thyroid disorder			0.892
Yes	2 (7.7)	24 (92.3)	
No	250 (7.0)	3,316 (93.0)	
Allergy/Asthma			0.407
Yes	1 (3.2)	30 (96.8)	
No	251 (7.1)	3,310 (92.9)	
Vaccination against influenza			0.012
Yes	41 (10.6)	344 (89.4)	
No	211 (6.6)	2,995 (93.4)	

^1^This column includes patients with COVID-19 with a positive PCR result and patients whose disease was confirmed only by observing symptoms.

**Table 3:** The breakthrough infection among different COVID-19 vaccines.

Variable	COVID-19 vaccine, *n* (%)					*p*-value
	Gam-COVID-Vac	ChAdOx1-S	BIBP-CorV	BBV152	All vaccines	
Number of vaccines administrated	1,181 (32.9)	916 (25.5)	1,418 (39.5)	76 (2.1)	3,591 (100)	–
History of COVID-19 infection	340 (32.6)	274 (26.3)	404 (38.8)	24 (2.3)	1,042 (29.0)	0.586
Breakthrough infection (PCR positive)	37 (22.8)	57 (35.3)	60 (37.0)	8 (4.9)	162 (4.5)	0.003
ICU admission/death	0 (0)	0 (0)	0 (0)	0 (0)	0 (0)	–
Moderate - hospitalized	3 (21.4)	0 (0)	11 (78.6)	0 (0)	14 (0.4)	–
Moderate - outpatient	22 (31.9)	22 (31.9)	23 (33.3)	2 (2.9)	69 (1.9)	0.400
Mild	12 (16.0)	34 (45.3)	26 (34.7)	3 (4)	75 (2.1)	0.001
Breakthrough infection (Suspicious for COVID-19)	56 (22.8)	91 (37.0)	93 (37.8)	6 (2.4)	246 (6.9)	0.001
ICU admission/death	0 (0)	0 (0)	0 (0)	0 (0)	0 (0)	-
Moderate - hospitalized	4 (19.3)	3 (14.2)	13 (61.8)	1 (4.7)	21 (0.6)	0.079
Moderate - outpatient	25 (24.0)	40 (38.5)	37 (35.6)	2 (1.9)	104 (2.9)	0.007
Mild	27 (22.3)	48 (39.7)	43 (35.5)	3 (2.5)	121 (3.4)	0.001
During immunization (PCR positive)	6 (16.6)	20 (55.6)	10 (27.8)	0 (0)	36 (1.0)	0.001
During immunization (Suspicious for COVID-19)	7 (10.6)	46 (69.7)	10 (15.2)	3 (4.5)	66 (1.8)	0.001

“During immunization” is a period from the first dose to 14 days after receiving the second dose. “After immunization” is a period from 14 days after receiving the second dose to a follow-up call, equivalent to a breakthrough infection. PCR, polymerase chain reaction, ICU, intensive care unit, NA, not applicable.

Moreover, no comorbidities were observed in these participants. The age range of this group was between 30 and 47 years and included 9 females (56%). No severe infection was evident in the studied population.

**Figure 2:**
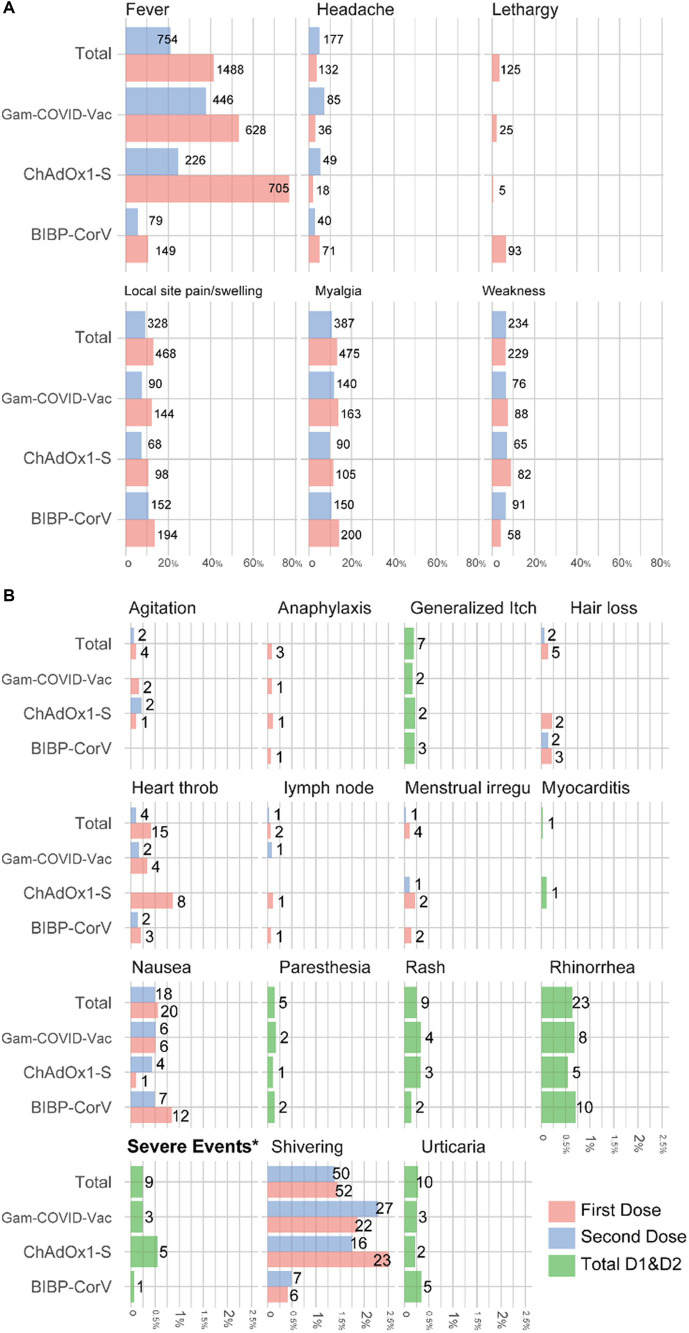
Percentage and number of adverse events among medical students who received two doses of COVID-19 vaccine, stratified by type of vaccine **(A)** more frequent adverse reactions; **(B)** rare adverse reactions.

Figure 2 shows the number of adverse reactions that occurred after each dose of the vaccine in the studied subjects. Also, the number of people who experienced adverse reactions within a week of the first or second dose of the vaccine is presented in Table 4. Fever (45.9%), Local site pain and swelling (16.8%), myalgia (16.4%), and weakness (10.1%) are, respectively, the most common adverse reactions observed within 1 week after receiving the vaccine. Complications such as myocarditis (0.03%), paresthesia (0.14%), hair loss (0.14%), and heartthrob (0.5%) were also observed with smaller amounts in the study subjects. Severe complications occurred in 9 participants (Anaphylaxis: 3, Rash: 1, heartthrob: 2, severe weakness, and fever: 4).

**Table 4:** Medical students experiencing an adverse reaction within 1 week of COVID-19 vaccination.

Adverse reaction	COVID-19 vaccine, *n* (%)						*p*-value
	BIBP-CorV	Gam-COVID-Vac	ChAdOx1-S	COVIran Barekat	BBV152	All vaccines	
Local site pain and swelling	267 (44.4)	183 (30.5)	115 (19.2)	28 (4.6)	8 (1.3)	601 (16.7)	< 0.0001
Lethargy	93 (74.4)	25 (20)	5 (4)	2 (1.6)	0 (0)	125 (3.4)	0.004
Fever	594 (36.0)	584 (35.4)	441 (26.7)	19 (1.2)	11 (0.7)	1,649 (45.9)	0.001
Headache	45 (34.1)	29 (22.0)	58 (43.9)	0 (0)	0 (0)	132 (3.7)	< 0.0001
Shivering	33 (38.8)	20 (23.5)	32 (37.6)	0 (0)	0 (0)	85 (2.4)	0.057
Nausea	10 (30.3)	13 (39.4)	10 (30.3)	0 (0)	0 (0)	33 (0.9)	0.711
Menstrual irregularity	2 (40.0)	0 (0)	3 (60.0)	0 (0)	0 (0)	5 (0.1)	0.394
Myalgia	193 (32.8)	196 (33.3)	183 (31.2)	9 (1.5)	7 (1.2)	588 (16.4)	0.001
Agitation	0 (0)	2 (33.3)	3 (50.0)	0 (0)	1 (16.7)	6 (0.2)	<0.0001
Weakness	113 (31.0)	131 (36.3)	116 (32.0)	0 (0)	3 (0.7)	363 (10.1)	<0.0001
Heartthrob	6 (33.4)	4 (22.2)	8 (44.4)	0 (0)	0 (0)	18 (0.5)	0.445
Hair loss	3 (60.0)	0 (0)	2 (40.0)	0 (0)	0 (0)	5 (0.1)	0.610
lymph node	1 (33.3)	1 (33.3)	1 (33.3)	0 (0)	0 (0)	3 (0.1)	0.997
Anaphylaxis	1 (33.3)	1 (33.3)	1 (33.3)	0 (0)	0 (0)	3 (0.1)	0.997
Rhinorrhea	10 (43.5)	8 (34.8)	5 (21.7)	0 (0)	0 (0)	23 (0.6)	0.948
urticaria	5 (50.0)	3 (30.0)	2 (20.0)	0 (0)	0 (0)	10 (0.3)	0.959
Paresthesia	2 (40.0)	2 (40.0)	1 (20.0)	0 (0)	0 (0)	5 (0.1)	0.993
Generalized Itch	3 (42.8)	2 (28.6)	2 (28.6)	0 (0)	0 (0)	7 (0.2)	0.994
Myocarditis	0 (0)	0 (0)	1 (100)	0 (0)	0 (0)	1 (0.03)	0.571
Rash	2 (22.2)	4 (44.4)	3 (33.4)	0 (0)	0 (0)	9 (0.3)	0.835
Severe events (needing hospitalization)	1 (11.1)	3 (33.4)	5 (55.5)	0 (0)	0 (0)	9 (0.3)	0.265

## Discussion

Newly emerged COVID-19 variants are of great concern since they can be highly transmissible, severe, or fatal. The mutations can also help evade detection or reduce the effectiveness of treatments or vaccines (1). Vaccination during the COVID-19 pandemic is the only known effective means to prevent COVID-19 spread and prospect mutations; however, any fully vaccinated subject may experience COVID-19 infection. Thus, understanding the complications and effectiveness of the available vaccines will aid us in developing better policies. As far as we know, no study has investigated the breakthrough infection of COVID-19 among medical and paramedical students, so this study was conducted on healthcare students more exposed to COVID-19 infection. The study was performed during the most violent peak of COVID-19 in Summer in Iran, with 30,519 reported fatalities and 71,297 excess deaths (31), while only 16.2% of the general population were fully vaccinated by the end of summer (32).

Among the 3,591 medical and paramedical students, our study reported a PCR-confirmed vaccine breakthrough of 5.13%, significantly higher than most previous studies. For example, in the last survey conducted by Bergwerk on vaccinated health workers, 2.6% of the participants in the study had a breakthrough COVID-19 infection (33). Also, in a systematic survey conducted on 651,595 people vaccinated with two doses, breakthrough infection was observed in 3.95% of the studied people (34). The high exposure risk of participants, low vaccine coverage, administrated types of vaccines, dominance of delta variants, or coincidence with the pandemic wave may explain this high vaccine breakthrough infection rate to some extent.

This study was performed on the earliest fully vaccinated population and coincided with the emergence of the Delta variant and the most devastating COVID-19 waves in Iran. The estimated initiation time of the follow-up period was mid-April when only 0.12% of the national population was fully vaccinated (35). At that time, countries with mostly unvaccinated populations (such as Iran and India) experienced a brutal coronavirus wave. As in a single-center study in India, 13.3% of healthcare workers experienced breakthrough infections during a massive COVID-19 wave with the Alpha and Delta dominancy period (2). At the time of Delta variant dominancy in the USA, when 50.2% of the population was immunized, the weekly incidence rate of breakthrough infections rose from 6.94 to 130.23 per 100,000 fully vaccinated (36). Therefore, the effect of the Delta variant on delineating vaccine effectiveness is indisputable (4, 24). Although not enough data is available regarding the share of variants in Iran, the immune escape by Delta variant may play a role in the high breakthrough infection rate in countries with low vaccination rates.

In the latest official available data, the efficacy of Gam-COVID-Vac, ChAdOx1-S, and BIBP-CorV were reported to be 91.6, 72, and 79%, respectively (10, 37, 38). Our study observed the lowest breakthrough infection rate in participants vaccinated with Gam-COVID-Vac. Vaccines have been proven to prevent contracting severe COVID-19 effectively. Despite administering the BIBP-CorV vaccine to less exposed healthcare students, ChAdOx1-S and Gam-COVID-Vac significantly prevented severe COVID-19. In addition, COVID-19 infection after the first dose was significantly more prevalent among participants vaccinated with ChAdOx1-S than among those who received other vaccines. This can be justified by the long interval between its first and second dose (8 to 12 weeks for ChAdOx1-S relative to 4 weeks for other vaccines). The best vaccine in pandemic waves should be decided on the basis of the efficacy and shortest time to reach maximum effectiveness.

Recent studies suggested a history of COVID-19 infection and healthcare jobs as factors for breakthrough infections. A systematic review mentioned that most COVID-19 re-infections occur in healthcare workers because of their prolonged exposure (34). Vaccine breakthrough was less common in our participants experiencing COVID-19 infection before vaccination, which aligns with previous studies (39). In our study, about 29% of the participants had a history of COVID-19 infection, yet the breakthrough infection rates were high. A survey of 126,586 vaccine recipients reported that working in healthcare centers is associated with a higher risk of breakthrough infection (40). Although exposure is essential, our study’s rate of 5.13% breakthrough cases is very high compared to previous studies on healthcare workers (33, 40).

Since no study has assessed the side effects of different types of vaccines in Iran, we also studied this issue. The most commonly reported side effects were fever, lethargy/fatigue, weakness, myalgia, and pain at the injection site. Inconsistent with previous studies, vaccines without adjuvant (Gam-COVID-Vac and ChAdOx1-S) were accompanied by a higher incidence of systemic adverse events, while vaccines with excipients consisting of Aluminum compounds (COVIran Barekat, BBV152, and BIBP-CorV) caused local reactions (41). In our study, fever was the most reported adverse event of the COVID-19 vaccine and most probable in the first dose administration. Severe post-vaccination adverse events, including anaphylaxis, rash, heartthrob, severe weakness, and fever, were present in 0.25% (*n* = 9) of our population, in which ChAdOx1-S and Gam-COVID-Vac were more likely to develop such events. Severe weakness and fever had the highest number of severe side effects among the serious side effects investigated in our study. Also, the ChAdOx1-S vaccine caused the highest number of severe side effects among the examined vaccines. According to the survey conducted by Huh et al., the incidence of anaphylaxis associated with vaccination increases with time, and according to another study conducted in July 2021, only one case of acute allergic reaction with shortness of breath and hypotension following vaccination was reported (42). In our study, 3 people who received the vaccine had anaphylactic shock. A previous report on 1954 healthcare workers showed a 0.4% (*n* = 9) severe post-vaccination adverse incident leading to hospital attendance; BIBP-CorV (*n* = 1), Gam-COVID-Vac V (*n* = 3), and ChAdOx1-S (*n* = 5) (43). For this reason, it is vital to constantly monitor side effects to create appropriate policies and inform the public. However, according to the results of our study and other studies that have investigated the side effects of vaccines and reported mild to moderate results in the side effects, the occurrence of such side effects during immunization can be considered acceptable (44–46).

This study investigated breakthrough infection and adverse events at young ages. There are some significant limitations to this study. This was a retrospective study with no control group, and investigation of vaccine effectiveness was impossible. In addition, this study did not include asymptomatic COVID-19 breakthrough infections; hence, underestimating breakthrough infection cases is possible. Comparison between the types of vaccines should be avoided since the time and administration settings were not similar. At first, Gam-Cov-Vac and ChAdOx1-S were more available for administration to front-line medical interns, whereas BIBP-CorV was mainly administered to students with less exposure. Also, our study may miss a fatality ratio because of its retrospective nature. However, we checked authorities for any mortality report found to be zero. Further prospective cohorts and active surveillance programs are warranted to investigate complications of COVID-19 vaccines.

## Conclusion

Vaccination prevented severe COVID-19 infection, although a high breakthrough infection rate was evident among Iranian medical students during the Delta variant’s peak. These data show that the effectiveness of different vaccines may be different and even fragile during emerging new variants and in high-exposure settings. In this way, to fully control COVID-19 infection, in addition to the continuation of simple preventive actions such as wearing a face mask and social distancing, a continuous assessment of the immunization of vaccines in the long term and against new species should be done. In addition, we observed that adverse events are rare, and the benefits of vaccination outweigh the side effects. However, constant monitoring of side effects after immunization is crucial. However, many limitations challenged this study, and the results should be cautious.

## Data availability statement

The raw data supporting the conclusions of this article will be made available by the authors, without undue reservation.

## Ethics statement

The studies involving humans were approved by the Institutional Review Board of Shahid Beheshti University of Medical Sciences. The studies were conducted in accordance with the local legislation and institutional requirements. The participants provided their written informed consent to participate in this study.

## Author contributions

AAl: Methodology, Project administration, Writing – original draft. SS-N: Data curation, Formal analysis, Writing – original draft. ZS: Data curation, Writing – original draft. AE: Data curation, Writing – original draft. HN: Data curation, Visualization, Writing – original draft. MH: Writing – original draft, Writing – review & editing. MMo: Investigation, Writing – original draft. PK: Data curation, Investigation, Writing – original draft. MI: Data curation, Investigation, Writing – original draft. HA: Data curation, Investigation, Writing – original draft. AAm: Data curation, Investigation, Writing – original draft. HM: Data curation, Investigation, Writing – original draft. AC: Data curation, Investigation, Writing – original draft. AM: Data curation, Investigation, Writing – original draft. MS: Data curation, Investigation, Writing – original draft. AN: Data curation, Investigation, Writing – original draft. MMa: Data curation, Investigation, Writing – original draft. AS: Data curation, Investigation, Writing – original draft. SS: Data curation, Investigation, Writing – original draft. SK: Data curation, Investigation, Writing – original draft. PM: Data curation, Investigation, Writing – original draft. MaM: Data curation, Investigation, Writing – original draft. SA: Data curation, Investigation, Writing – original draft. NA: Data curation, Investigation, Writing – original draft. SD: Data curation, Investigation, Writing – original draft. AT: Data curation, Writing – review & editing. MB: Methodology, Validation, Writing – review & editing. ME: Methodology, Supervision, Writing – review & editing.
